# Musical Auditory Stimulation Influences Heart Rate Autonomic Responses to Endodontic Treatment

**DOI:** 10.1155/2017/4847869

**Published:** 2017-01-15

**Authors:** Milana Drumond Ramos Santana, Eli Carlos Martiniano, Larissa Raylane Lucas Monteiro, Vitor E. Valenti, David M. Garner, Isabel Cristina Esposito Sorpreso, Luiz Carlos de Abreu

**Affiliations:** ^1^Laboratório de Delineamento de Estudos e Escrita Científica, Faculdade de Medicina do ABC, Santo André, SP, Brazil; ^2^Faculdade de Juazeiro do Norte, Juazeiro do Norte, CE, Brazil; ^3^Centro de Estudos do Sistema Nervoso Autônomo (CESNA), Departamento de Fonoaudiologia, Faculdade de Filosofia e Ciências, UNESP, Marília, SP, Brazil; ^4^Cardiorespiratory Research Group, Department of Biological and Medical Sciences, Faculty of Health and Life Sciences, Oxford Brookes University, Gipsy Lane, Oxford OX3 0BP, UK; ^5^Disciplina de Ginecologia do Departamento de Obstetrícia e Ginecologia, FMUSP, São Paulo, SP, Brazil

## Abstract

We aimed to evaluate the acute effect of musical auditory stimulation on heart rate autonomic regulation during endodontic treatment. The study included 50 subjects from either gender between 18 and 40 years old, diagnosed with irreversible pulpitis or pulp necrosis of the upper front teeth and endodontic treatment indication. HRV was recorded 10 minutes before (T1), during (T2), and immediately (T3 and T4) after endodontic treatment. The volunteers were randomly divided into two equal groups: exposed to music (during T2, T3, and T4) or not. We found no difference regarding salivary cortisol and anxiety score. In the group with musical stimulation heart rate decreased in T3 compared to T1 and mean RR interval increased in T2 and T3 compared to T1. SDNN and TINN indices decreased in T3 compared to T4, the RMSSD and SD1 increased in T4 compared to T1, the SD2 increased compared to T3, and LF (low frequency band) increased in T4 compared to T1 and T3. In the control group, only RMSSD and SD1 increased in T3 compared to T1. Musical auditory stimulation enhanced heart rate autonomic modulation during endodontic treatment.

## 1. Introduction

Dental treatment has been reported by many patients as a situation generating stress and anxiety. One of the main elements that seem to interfere with the behavior of most individuals seeking dental care is the acceptance that they will be subjected to some kind of discomfort during the treatment [1–4].

In this context, the literature has investigated alternative therapies to improve stressful situations [5, 6]. Among alternative therapies, we may include the beneficial actions of musical therapy, which have been demonstrated as an alternative therapy for cardiovascular syndromes [7]. The use of relaxant music reduces the levels of heart rate, systolic blood pressure, and diastolic blood pressure [8].

Stress and anxiety are associated with the regulation of cardiac function by the autonomic nervous system (ANS), which allows us to evaluate the balance between the sympathetic and parasympathetic systems. In the heart, the ANS specifically influences heart rate and cardiac contractility. Furthermore, to increase or decrease of heart rate, ANS also regularly controls heart beats. The fluctuation of consecutive heart beats is analyzed through heart rate variability (HRV) [9–12].

Along these lines, the development of technical, scientific, and biological procedures in endodontic treatment achieves highly successful results. However, in spite of this, the treatment involves several stages ranging from anesthesia until obliteration of the root canal system. Also, the technique can be accomplished by basic technical operative steps which are prone to failure and various complications in its clinical progression [13].

Consequently, we believe that, during endodontic treatment, musical auditory stimulation may improve HRV. Knowledge of physiological responses involved in endodontic treatment is imperative because it provides a more appropriate treatment plan to the patient and provides greater safety clinically for the endodontist. Analysis of the ANS oscillations during endodontic procedure provides information, which can cause potential risks for cardiovascular system. Therefore, we investigated the acute effects of musical auditory stimulation on heart rate autonomic regulation during endodontic treatment.

## 2. Method

### 2.1. Population Study

Fifty subjects participated in this study with 13 females and 12 males in the music group and 12 females and 13 males in the control group. All participants aged between 18 and 40 years were examined. Everyone was diagnosed with irreversible pulpitis or pulp necrosis of the upper front teeth and endodontic treatment indication. The patients were divided into two equal groups: control group (patients who were submitted to standardized endodontic treatment) and music group (patients who were submitted to standardized endodontic treatment and were exposed to musical auditory stimulation). The sample was selected from endodontic treated patients undergoing the root canal procedure. All volunteers were informed about the procedures and objectives of the study and, after agreeing, they signed an informed consent form which was confidential. The study was approved by the Ethics Committee in Research of our University (Protocol number 993.350).

### 2.2. Exclusion Criteria

We considered the following as exclusion criteria: smokers; individuals with cardiorespiratory, neurological disorders and other reported impairments that prevented the subject from performing the procedures and treatment with drugs that influence cardiac autonomic regulation; patients who have undergone previous root canal treatment; women between 10–15 and 20–25 days after the first day of the menstrual cycle in order to avoid influence of luteal and follicular phase; pregnant women; and subjects who reported hearing disorders.

### 2.3. Initial Evaluation

Prior to the endodontic treatment the following information was collected: age, gender, mass, height, and body mass index (BMI). Anthropometric measurements were obtained according to the recommendations described by Lohman [14]. Mass was measured on a digital scale (W 200/5, Welmy, Brazil) with a precision of 0.1 kg and height using a stadiometer (ES 2020 Sanny, Brazil). BMI was calculated using the following formula: mass (kg)/height (m)^2^.

### 2.4. Experimental Protocols

Data collection was undertaken in a room with temperature between 21°C and 25°C and humidity between 50% and 60%. The volunteers were instructed to not ingest alcohol, caffeine, or other ANS stimulants for 24 hours before the evaluation. The volunteers were instructed to remain at rest and avoid talking during the collection.

Primarily, the blood pressure (BP) measurement was implemented. Subsequently, the saliva collection for salivary cortisol measurement was undertaken. Following this the analysis of the protocol of cardiac modulation during endodontic treatment was assumed.

### 2.5. Blood Pressure Measurement

Systolic (SBP) and Diastolic Blood Pressure (DBP) measurements were performed using an aneroid sphygmomanometer (Tycos®) and stethoscope (Sprague®). BP was measured before and after the endodontic treatment.

### 2.6. Measurement of Salivary Cortisol

We did not induce salivation to collect saliva samples; we collected salivary cortisol samples before and after endodontic treatment between 14:00 and 17:00 by the same researcher. The volunteers were told to not eat or drink one hour before collecting saliva and wash their mouths with water. The Salivette® tubes were refrigerated until analysis of the material.

Data assembly was performed using a Salivette tube consisting of a plastic tube that contains a highly absorbent cotton roll, maintained in the mouth for about three minutes. Then, it was placed inside the collector plastic tube. After collecting all the Salivette tubes, samples were centrifuged for 20 minutes at 2800 rpm at a temperature of 4°C. During centrifugation, saliva passed the cylindrical swab medium through the cavity in the bottom of the drop tube to the clean centrifuge tube. Mucus and airborne particles were captured in the conical tip of the tube, allowing for easy decanting of clarified saliva.

Following centrifugation, the saliva was transferred to an Eppendorf type tube with lid and stored in a freezer at −20°C until the time when it was inspected. The material was cooled, ensured by the use of dry ice, Styrofoam, and a thermal bag. The samples were processed using the electrochemiluminescence method.

### 2.7. HRV Analysis during Endodontic Treatment

HRV was recorded in the first session of endodontic treatment at four intervals:

- 10 minutes before the beginning of root canal therapy session (T1)
- The initial 10 minutes after the application of local anesthetic (T2)
- The total duration of endodontic treatment (T3)
- 30 minutes after the endodontic treatment termination (T4)

After the initial assessment, we placed the heart rate receiver Polar RS800CX (Polar Electro, Finland) on the chest of the volunteers at the distal third of the sternum region and strapped at the wrist. After placing the strap and the monitor, the protocol was initiated.

### 2.8. Protocol of Endodontic Treatment

The endodontic treatment was performed by the same dentist. We describe below all the steps of endodontic treatment:

- Inferior alveolar nerve anesthesia (2% DFL® ALPHACAINE)
- Decayed tissue removal and access to the pulp chamber (spherical diamond burs numbers 1012, 1014, and 1015, KG Sorensen®) compatible with the size of the pulp chamber, mounted on high speed turbine, air-cooled water, and Endo Z bur (Les Fils d' August Mailefer SA)
- The operating field isolation with rubber dam
- Chemical-mechanical preparation of root canals (Hand files Flexofile Dentsply® and sodium hypochlorite 2.5%)
- Channel drying (Dentsply paper cones)
- Intracanal medication (SS White®)
- Cotton ball laying on channel input
- Interim sealing (IRM®)

### 2.9. The Effect of Music on HRV Analysis

The volunteers were randomly divided into two equal groups (*n* = 25), in which a group performed endodontic treatment while listened to music during T2, T3, and T4 periods and the other group was not exposed to musical auditory stimulation. The music played was “Träumerei” of Kinderszenen Op. 15-7 and was conveyed through a mp4 player (Sony® NWZ-E473) to an earphone. The control group remained with the earphone turned-off.

### 2.10. HRV Analysis

HRV analysis followed guidelines from the aforementioned Task Force [15]. The RR intervals were recorded by the portable RS800CX heart rate (HR) monitor with a sampling rate of 1000 Hz. They were then downloaded to the Polar Precision Performance program (v. 3.0, Polar Electro, Finland). The software enabled the visualization of HR and the extraction of a cardiac period (RR interval) file in “txt” format. Subsequently, digital filtering was complemented with manual filtering for the elimination of premature ectopic beats and artefacts; the final 256 RR intervals of each period were applied for the data analysis, because this was the period in which the subjects were stable.

Digital filtering was based on an algorithm in the Polar Protrainer software (Polar Electro, Finland). The algorithm intends to detect all measurement errors through analyzing median and moving average based filtering methods. The criteria adapt to the RR interval to be verified; if RR interval is very stable, relatively small peaks will be interpreted as errors. However, if RR interval includes very large variability, the criteria will be looser. Very small errors are not corrected. In the correction phase, the algorithm computes several more matching “candidates” to substitute the detected errors. The algorithm identifies the difference between previous and the next accepted RR interval and then makes the series of corrected values to follow the same differential coefficient. The special property of the error correction algorithm maintains the total time of the recording: that is, the sum of RR intervals is exactly the same as the elapsed time (http://support.polar.com/en/support/tips/How_R-R_Data_is_Filtered).

Manual filtering was performed by using Microsoft® Excel® 2013, it was based on visual inspection that detect artefacts and ectopic beats.

Only series with more than 95% sinus rhythm were included in the study; we excluded records with a percentage of artefacts higher than 5% [11, 16]. For calculation of the linear indices we applied the HRV analysis software (Kubios HRV® v.1.1 for Windows, Biomedical Signal Analysis Group, Department of Applied Physics, University of Kuopio, Finland) [17] (see Supplement files).

### 2.11. Geometric Indices of HRV

The geometric indices of HRV included quantitative analysis of the Poincaré plot (SD1: standard deviation of the instantaneous variability of the beat-to-beat heart rate; SD2: standard deviation of long-term continuous RR interval variability and SD1/SD2 ratio) and time domain RRtri (triangular index) and TINN (triangular interpolation of RR intervals) indices. Significance of the abovementioned indices was previously described [15, 18].

## 3. Statistical Analysis

To determine the sample size a priori knowledge was needed, based on Moreno et al. [19] findings for SDNN (standard deviation of all normal RR intervals), which represents the activity of both ANS branches, indicating the overall behavior of HRV [11]. A sample size of 18 participants per group was stipulated by a test of hypothesis (two-tailed), with 5% level of significance and 80% power.

For comparison between individuals of the same group, it was necessary to assess data normality by the Shapiro-Wilk test. When normal distribution was achieved, we applied the unpaired Student's* t*-test and ANOVA for repeated measures followed by Bonferroni posttest. In circumstances where the normal distribution was unaccepted, the Mann–Whitney test and the Friedman test followed by Dunn's posttest was applied. Dissimilarities in these tests were considered statistically significant when the value of “*p*” is less than 0.05. The statistical program applied was GraphPad Software StatMate 2:00 version for Windows, GraphPad Software, San Diego, California, USA.

## 4. Results

After applying the normality test we checked that all distributions evaluated were parametric. In this sense, data were presented as mean and standard deviation.

Table 1 displays anthropometric data including age, height, mass, and BMI of volunteers from both groups.

In the control group, we noted that RR interval was increased during endodontic treatment (T3) compared to before endodontic procedures (T1). RMSSD (root-mean square of differences between adjacent normal RR intervals) and SD1 indices were also increased during endodontic treatment (T3) compared to before endodontic procedures (T1) (Table 2).

Regarding the groups subjected to musical auditory stimulation during the endodontic treatment, we observed that DAP increased after the procedures (T4). HR decreased while RR interval decreased during endodontic treatment (T3) compared to before treatment (T1) (Table 3).

Regarding HRV analysis, SDNN, TINN, and SD2 indices were weakened during endodontic treatment (T3) compared to after treatment (T4). Moreover, RMSSD and SD1 were increased after treatment (T4) compared to before treatment (T1) (Table 3).

Regarding spectral analysis, LF in absolute units was increased after treatment (T4) compared to before (T1) and during treatment (T3) (Table 3).

In our study, we found a decrease in the SDNN, SD2, and LF indices during endodontic treatment with musical stimulation compared to after treatment. Those indices correspond to overall HR modulation [15].

We also compared HRV indices between both groups at all instants. SDNN (*p* = 0.01), TINN (*p* = 0.01), and SD2 (*p* = 0.002) were increased in the control group during T3 with no changes in T1, T2, and T4. TINN was higher in T1 (*p* = 0.04) and T3 (*p* = 0.001) in the control group with no changes in T2 and T4. The LF/HF ratio was increased in T1 (*p* = 0.04) and T2 (*p* = 0.02) in the control group with no changes in T3 and T4.

There was no significant difference between groups for mean RR interval, RMSSD, pNN40, SD1, LF (ms^2^), LF (nu), HF (ms^2^), and HF (nu) LF/HF ratio during T1, T2, T3, and T4.

## 5. Discussion

This study assessed the acute effect of musical auditory stimulation on HRV and salivary cortisol during endodontic treatment. Accordingly, it was found that the musical auditory stimulus improved heart rate autonomic responses induced by endodontic treatment. There were no significant changes in cortisol levels, which indicates the involvement of ANS on this mechanism.

During endodontic treatment, the patient is commonly presented in a stressful condition [1]. The physical or psychological stress stimulates the hypothalamus to synthesize corticotrophin releasing hormone, which then stimulates the pituitary gland into synthesizing the adrenocorticotropic hormone, which flows via the bloodstream to the adrenal glands and stimulate the synthesis of cortisol, the “stress hormone” [4]. Simultaneously there is activation of the sympathetic nervous system and this releases catecholamines (norepinephrine and epinephrine), which then trigger increased HR, myocardial contraction, cardiac output, and arterial pressure [20].

Equally, we failed to find significant changes in cortisol levels after endodontic treatment in control and music group. Nevertheless, there is a trend of declination after treatment in the group exposed to musical auditory stimulation.

Regarding the effects of stress, anxiety, and depression on modulation of the ANS, Langewitz et al. [21] evaluated the effect of mental stress on HRV and they observed a decrease of HRV. According to Morino et al. [22], the HRV spectral analysis is advantageous in the evaluation of tension and anxiety.

The study of Matsumura et al. [23] assessed changes in blood pressure and HRV during dental surgery. It was verified that elderly patients had a further severe increase in blood pressure during dental surgeries compared to younger patients and that HRV during dental surgery differs between younger and elderly patients. Miura et al. [12] evaluated the changes in blood pressure, pulse rate, and HRV during dental surgery in hypertensive patients. The authors concluded that suppression of cardiac sympathetic nervous system during dental surgery may weaken responses in patients with hypertension.

Moreover, Santana et al. [24] observed that during endodontic treatment HRV is diminished. Furthermore, after application of the local anesthetic the HRV increases due to cardiac parasympathetic predominance. Like this, we aimed to verify the effects of an alternative therapy to improve HRV responses to endodontic treatment.

In our study, we found a decrease in the SDNN, SD2, and LF indices during endodontic treatment with musical stimulation compared to after treatment. Those indices correspond to overall HR modulation [15].

The auditory stimulation with music influences the cardiovascular system, since there is a correlation between the noise intensity and sympathovagal balance. It is hypothesized that dopamine released in the striatal system induced by melodic and cheerful songs is involved in autonomic regulation [7, 25]. In this way, music is capable of improving autonomic regulation of heart rate.

Conferring to da Silva et al. [26] classical music increases the parasympathetic modulation. Riganello et al. [27] evaluated the HRV and its correlation with musical stimulation of different complexities in healthy subjects and with disorders of perception. The results illustrated that the musical stimulus improved the autonomic response of patients with perception disorders.

Some studies illustrated a correlation between parasympathetic heart rate regulation (HF: high frequency band) and music [28, 29]. Supplementary studies suggested that the music decreases excitatory activation of the parasympathetic nervous system in healthy subjects [28] and heavy metal sharply decreases HRV [23]. do Amaral et al. [25] described that auditory stimulation with baroque music did not affect heart rate control. Yet, higher sound intensity of heavy metal reduced the global component of HRV.

Also, reduction of the LF index was observed in the study of da Silva et al. [26], which evaluated the acute effects of heavy metal and baroque musical auditory stimulation on HRV in healthy males. The authors noted that the LF band, in both absolute and normalized standard units, was lessened during exposure to both styles of music compared to the control groups. In the same study, there was a reduction in SDNN index during heavy metal music.

We reported that the TINN index was also decreased during endodontic treatment in the music group. Previously a study [25] illustrated that auditory stimulation with heavy metal music caused a reduction of TINN index in females. Nevertheless, in another study [30] no change was observed in relation to TINN during the musical stimulation with heavy metal in males.

Founded on our data, RMSSD and SD1 indices increased after endodontic treatment compared to before treatment. Our data points to a beneficial elevation in parasympathetic heart rate regulation induced by music during endodontic treatment. Ferreira et al. [31] testified increases in SDNN, RMSSD, pNN50 (percentage of adjacent RR intervals with a difference of duration greater than 50 ms), and LF (ms^2^) after stimulation with heavy metal music in healthy females. The authors also found that SDNN, LF (ms^2^ and nu), and the LF/HF ratio increased while the HF index (nu) decreased after exposure to classical baroque music. The abovementioned study supports our results.

In this study, there was deficiency of significance of salivary cortisol before and after endodontic treatment. In this context, we indicate that music operated through ANS and not via hypothalamic-pituitary axis due to the short period of endodontic treatment. Nonetheless, there was a trend (*p* = 0.0577) to reduce cortisol levels in the music group. Hence, we suggest that a more prolonged time of exposure to music may influence cortisol levels.

Previously, a study stated that music has a beneficial effect in controlling anxiety to dental treatment. The authors found significant differences in salivary cortisol levels, systolic and diastolic pressure, HR, body temperature, and stimulated salivary flow for the group treated with musical treatment [32]. Likewise, our findings reinforce the beneficial use of music during endodontic treatment.

Mehr et al. [33] assessed techniques of music therapy in different dental specialties, wherein 81 patients reported the dental treatment as unpleasant and then were driven to a discrete music therapy. After each dental treatment session, both patients and the dentist responded to a questionnaire. In conclusion, the authors encouraged music therapy in dental treatment, which supports our findings here.

According to our results, we reported a significant statistical increase of LF and a simultaneous decrease of SDNN between T1 and T3 during auditory stimulus; it indicated that the music induced excitatory effects on HRV. A previous study assisted us to explain this response. Roque et al. [34] observed that relaxant baroque music slightly decreased global HRV. Taken together, we believe that the musical auditory stimulation used in our study may have caused a similar reaction on HRV.

Some facts are worth considering. The age range was wide (18 to 40 years). Conversely, exclusion criteria helped to ensure regulatory standards regarding the sample selection. Factors such as age, gender, anthropometric characteristics, and health conditions have been shown to influence physiological reactions [35]. We investigated the patients in the first session of endodontic treatment in order to avoid prior familiarity with the endodontic treatment.

## 6. Conclusions

Musical auditory stimulation acutely improved heart rate autonomic regulation during endodontic treatment. Our results encourage the use of relaxant music during endodontic treatment in order to lessen cardiovascular responses.

## Figures and Tables

**Table 1:** Age, height, mass, and BMI of volunteers.

Variable	Mean ± standard-deviation		*p*
	Control	Music	
Age (years) (min-max)	29.16 ± 6.99 (18–40)	28.61 ± 6.88 (19–40)	0.7628
Height (m) (min-max)	1.61 ± 0.08 (1.36–1.8)	1.6 ± 0.08 (1.47–1.72)	0.6512
Mass (kg) (min-max)	65.72 ± 12.03 (43.3–91.3)	63.53 ± 10.1 (56–91)	0.4704
BMI (kg/m^2^) (min-max)	26.14 ± 6.44 (18.59–47.43)	24.82 ± 3.85 (18.72–33.89)	0.6176

m: meters; kg: kilograms; BMI: height and body mass index; min-max: minimum-maximum.

**Table 2:** Mean and standard deviation of SBP, DBP, and cortisol in T1 and T4, heart rate (HR), RR interval, and linear indices in T1, T2, T3, and T4 in the volunteers without auditory stimulus.

Variable	T1	T2	T3	T4	Value *p*
SAP (mmHg)	117.2 ± 17.68	—	—	118.4 ± 17	0.7033
DAP (mmHg)	77.6 ± 12	—	—	79.2 ± 9.96	0.3075
Cortisol (ug/dl)	0.23 ± 0.13	—	—	0.4 ± 0.77	0.1334
HR (bpm)	90.5 ± 20.96	89.42 ± 29.93	78.81 ± 14.1	86.69 ± 30.35	0.1851
RR (ms)	695.98 ± 136.66^*∗*^	731.06 ± 184.17	786.92 ± 126.75	753.5 ± 183.29	*p* < 0.0001
SDNN (ms)	45.11 ± 17.44	45.78 ± 30.28	54.08 ± 25.87	44.86 ± 16.98	0.4717
RMSSD (ms)	27.68 ± 17^*∗*^	33.1 ± 24.54	44.76 ± 25.49	34.94 ± 22.62	0.0251
pNN50 (ms)	10.69 ± 13.3	15.86 ± 18.49	22.76 ± 17.95	17.4 ± 17.67	0.0620
RRtri (ms)	9.86 ± 4.83	9.41 ± 5.58	11.66 ± 6.39	9.76 ± 5.2	0.1472
TINN (ms)	183.8 ± 74.76	163.8 ± 101.79	212.2 ± 101.43	166 ± 73.47	0.0626
SD1 (ms)	19.66 ± 12.05^*∗*^	23.5 ± 17.39	31.8 ± 18.15	24.83 ± 16.06	0.0240
SD2 (ms)	59.78 ± 23.89	59.33 ± 40.21	68.92 ± 33.47	57.26 ± 21.31	0.6685
LF (ms^2^)	598.96 ± 499.28	608.12 ± 739.56	870.43 ± 915.79	872.06 ± 910.3	0.3427
LF (nu)	64.06 ± 20.13	62.27 ± 22.72	57.24 ± 16.34	62.69 ± 20.39	0.8741
HF (ms^2^)	459.37 ± 669.23	491.84 ± 706.79	784.87 ± 960.68	529.4 ± 631.87	0.1104
HF (nu)	35.78 ± 20.06	37.52 ± 22.64	42.56 ± 16.33	37.18 ± 20.34	0.4329
LF/HF	3.42 ± 4.02	3.66 ± 4.47	2.23 ± 3.26	3.08 ± 3.07	0.8741

^*∗*^ *p* < 0,05: versus T3. ms: milliseconds; bpm: beat per minute; mmHg: millimeters of mercury; ug/dl: micrograms per deciliter; nu: standard unit; ms^2^: absolute unity; SDNN: standard deviation of all normal RR intervals; pNN50: percentage of adjacent RR intervals lasting difference greater than 50 milliseconds; RMSSD: square root of the mean square of differences between adjacent normal RR intervals; LF: low frequency; HF: high frequency; LF/HF: ratio low frequency/high frequency; RRtri, triangular index; TINN, triangular interpolation of RR intervals; SDI: standard deviation of the instantaneous variability of the beat-to-beat heart rate; SD2: standard deviation of long-term continuous RR interval variability; SD1/SD2 ratio: ratio between the short- and long-term variation of the RR intervals.

**Table 3:** Mean and standard deviation of SBP, DBP, and cortisol in T1 and T4, heart rate, RR interval, and linear indices in T1, T2, T3, and T4 in subjects with auditory stimulus.

Variable	T1	T2	T3	T4	Value *p*
SAP (mmHg)	116.15 ± 14.71	—	—	117.3 ± 11.85	0.6085
DAP (mmHg)	78.46 ± 10.07	—	—	82.3 ± 7.64	0.0288
Cortisol (ug/dl)	0.27 ± 0.17	—	—	0.24 ± 0.15	0.0577
HR (bpm)	82.25 ± 13.08	79.3 ± 15.02	76.73 ± 12.09^*∗*^	114.55 ± 177.84	0.0066
RR (ms)	747.93 ± 125.7	787.08 ± 151.53^*∗*^	804.03 ± 135.94^*∗*^	779.63 ± 122.1	0.0014
SDNN (ms)	40.34 ± 14.47	45.2 ± 16.35	38.51 ± 16.23^*∗∗*^	46.9 ± 15.09	0.0237
RMSSD (ms)	34.66 ± 18.01^*∗∗*^	41.92 ± 22.53	37.13 ± 21.71	39.65 ± 19.15	0.0240
pNN50 (ms)	15.39 ± 16.05	21.69 ± 19.42	16.84 ± 18.3	18.09 ± 15.11	0.1033
RRtri (ms)	8.31 ± 2.48	8.53 ± 2.83	8.36 ± 3.09	8.7 ± 1.84	0.6246
TINN (ms)	152.3 ± 56.46	156.73 ± 66.75	138.46 ± 67.03^*∗∗*^	171.53 ± 53.41	0.0320
SD1 (ms)	24.67 ± 12.8^*∗∗*^	29.89 ± 16.08	26.45 ± 15.42	28.23 ± 13.61	0.0310
SD2 (ms)	51 ± 17.61	55.34 ± 19.97	46.95 ± 18.64^*∗∗*^	59.74 ± 17.94	0.0336
LF (ms^2^)	286 ± 61.04^*∗∗*^	576.88 ± 423.06	529 ± 592.94^*∗∗*^	850.92 ± 615.42	0.0004
LF (nu)	57.77 ± 16.33	54.25 ± 18.99	49.14 ± 23.26	60.19 ± 15.09	0.0982
HF (ms^2^)	470.57 ± 466.43	539.38 ± 431.14	471.26 ± 466.46	654.69 ± 647.14	0.3741
HF (nu)	41.94 ± 16.26	45.5 ± 18.95	50.56 ± 23.15	39.64 ± 15.07	0.1007
LF/HF	1.89 ± 1.66	1.73 ± 1.46	1.89 ± 2.45	2.1 ± 1.97	0.1391

^*∗*^ *p* < 0,05: versus T1; ^*∗∗*^*p* < 0,05: versus T4. ms: milliseconds; bpm: beat per minute; mmHg: millimeters of mercury; ug/dl: micrograms per deciliter; nu: standard unit; ms^2^: absolute unity; SDNN: standard deviation of all normal RR intervals; pNN50: percentage of adjacent RR intervals lasting difference greater than 50 milliseconds; RMSSD: square root of the mean square of differences between adjacent normal RR intervals; LF: low frequency; HF: high frequency; LF/HF: ratio low frequency/high frequency; RRtri, triangular index; TINN, triangular interpolation of RR intervals; SDI: standard deviation of the instantaneous variability of the beat-to-beat heart rate; SD2: standard deviation of long-term continuous RR interval variability; SD1/SD2 ratio: ratio between the short- and long-term variation of the RR intervals.
